# Investigation of the presence of Non-carious cervical lesions (NCCLs) in ancient adult skulls: analyzing data from prehistoric and historical samples through a systematic review and meta-analysis

**DOI:** 10.1186/s12903-024-04154-4

**Published:** 2024-03-22

**Authors:** Mario Dioguardi, Francesca Spirito, Eleonora Lo Muzio, Diego Sovereto, Andrea Ballini, Mario Alovisi, Giusi Antonia Toto, Lorenzo Lo Muzio, Michele Di Cosola

**Affiliations:** 1 Department of Clinical and Experimental Medicine, University of Foggia, Via Rovelli 50, Foggia, 71122 Italy; 2 Department of Surgical Sciences, Dental School, University of Turin, Turin, 10127 Italy; 3 Department of Humanities, Letters, Cultural Heritage, Educational Sciences, University of Foggia, Foggia, 71122 Italy

**Keywords:** NCCL, Abfraction, erosion, Abrasion, Dental wear, Ancient skulls

## Abstract

**Objectives:**

Historically, the prevalence of caries has undergone significant changes, particularly increasing with the industrialization of sugar consumption. When examining ancient populations, lower caries rates are discovered, attributed in part to dietary factors. These populations consumed abrasive foods, leading to occlusal wear and reduced non-axial occlusal forces, potentially influencing Non-Carious Cervical Lesions (NCCLs). Although some attribute NCCLs to abfraction, the mechanism remains debated. This systematic review aims to evaluate the presence of NCCLs in ancient populations, shedding light on the factors contributing to their occurrence.

**Materials and Methods:**

The present systematic review was registered on PROSPERO, and the manuscript was prepared following PRISMA guidelines.

**Results:**

After the literature search and article screening, data from 6 studies were included in the meta-analysis, with only 2 reporting NCCLs in ancient skulls, encopassing 17 subjects in 805 examined skulls, suggesting their presence even before the widespread use of toothbrushes. This finding indicates a potential etiopathogenic mechanism linked to abfraction, but the cause is complex and involves abrasive and erosive factors closely tied to dietary habits.

**Conclusions:**

In summary, NCCLs were present in ancient populations, albeit with a much lower prevalence. Their occurrence cannot be solely attributed to wear mechanisms but must be connected to abrasive factors related to diet or practices with religious and cultural significance, such as the use of labrets. Clinical relevance: Th the knowledge of NCCLs presence in acient sculls is crucial today for better understand the associated risk factors. In this context, the analysis of ancient skulls allows us to discern the role that tooth brushing and diet played in the formation of NCCLs, over the past century.

## Introduction

Caries has a multifactorial etiology in which the combination of three aspects, such as the presence of bacteria, dietary habits, and tooth [1], synergistically contribute to the onset and progression of caries [2]. The prevalence of untreated dental caries in permanent teeth, as indicated by the 2013 Global Burden of Disease study, approaches rates close to 35% across all age groups, with an estimated 90% of the population being affected [3]. However, a systematic review with meta-analysis conducted in 2022 revealed considerable variability in prevalence based on geographical areas. The estimated overall pooled prevalence of dental caries was found to be globally around 45%, with regional variations of 17% (Europe), 29% (North America), 42% (South America), 48% (Africa), and 53% (Asia-Oceania) respectively [4].

These findings, can be partially compared to the prevalence data of dental caries in historical European populations. A further systematic review conducted in 2022 [5], reported that approximately 10% of teeth and nearly half of individuals already suffered from dental caries during the Neolithic period. A dramatic increase in the prevalence of caries was observed during the early modern era, affecting up to 95% of individuals and 60% of teeth [5].

The significant increase in dental caries can be attributed to dietary transformations that occurred concurrently with the transition from an hunter lifestyle, to an agricultural practice. Throughout the Bronze Age, Iron Age, and High Middle Ages, a consistent rise in the prevalence of caries was observed, paralleling the expansion and continual development of agricultural practices. This phenomenon can be linked to the intensification of agricultural activities, leading to a distinct dietary shift characterized by an increased intake of fermentable carbohydrates.

Specifically, during the late Middle Ages and the early modern era, a sudden wave in the spread of caries occurred. This phenomenon can be elucidated by the introduction into the human diet of substances such as sucrose and other easily fermentable carbohydrates, contributing to a microbiota-friendly environment, thus significantly contributing to the rise of dental caries during these specific historical periods.

During the industrialized period, the dietary habits, with the consumption of sugared substances, established an increase in the availability and affordability of sugar, which made this condition more prevalent [6]. In fact, the frequency of dental caries in a Byzantine pediatric population (7th to 10th Centuries) at Yenikapı (Constantinople, Istanbul), revealed that the overall frequency of carious lesions in the sample was 2.2%, which, linked with low erosive tooth wear, indicated a fishing community [7].

These data, compared with a study on a modern population, showed a prevalence data of 10.8% (12–19 year-old school children, Ibadan, Nigeria) [8]. In a Roman-British adult population (late 3th /early 5th century), the percentage of carious lesions was found to vary from 9.3% [9], 11.3% [10] to 15% [11]. It’s noteworthy that the localization of these lesions was predominantly at the cervical margin rather than on the occlusal surface (in line with occlusal dental wear data), or interproximal surfaces, which are the most frequent localization sites in the modern population [11].

Caries pathology is not the sole cause of mineralized tooth substance loss; in fact, among these causes, were find tooth occlusal surface wear and Non-Carious Cervical Lesions (NCCLs), that can be attributed to three main etiopathological mechanisms kwow as 1) erosion, 2-)abrasion and 3-) abfraction [12].

The first two mechanisms, were defined during a meeting held in Frankfurt between the European Organization for Research on Dental Caries and the Cariology Research Group of the International Association for Dental Research (IADR) [13]. The objective of this meeting was to establish the terminology for erosive tooth wear and dental caries. Dental erosion was defined as the chemical loss of mineralized dental substance caused by exposure to acids not derived from oral bacteria [13]. Dental abrasion was defined as the physical loss of mineralized dental tissue caused by objects other than teeth [13]. In addition, tooth wear is also determined by dental attrition which is defined as the physical loss of mineralized tooth substance caused by tooth-to-tooth contact.

Regarding abfraction, the mechanism of action is subject to controversy, there is no complete agreement on its actual existence. The doubt primarily revolves around the role that non-axial occlusal forces would play on the tooth’s surface [14].

In 1991, Grippo [14], utilize this term to define the biomechanical (physiochemical) effects of occlusal load, which induce tooth deformation and flexion, resulting in the loss of tooth tissue. This centralizes the role of occlusal load in the formation of a specific type of NCCLs, typically wedge-shaped with sharp margins, more frequently affecting premolars [15].

There are several indications supporting the theory of non-axial occlusal forces as contributors to certain NCCLs, characterized by parafunctions such as bruxism [16], which exhibits wear facets. Additionally, the occurrence of these lesions seems to be associated with patients lacking canine disclusion (77.2%) [17]. Furthermore, dental mobility appears to not predispose to NCCLs (1.9% of teeth with mobility) [17].

If the primary etiological cause of NCCLs is represented by abnormal occlusal loads, recognizing the primary mechanism as the phenomenon described as abfraction, the presence of these characteristic wedge-shaped lesions should also be found in populations predating the widespread adoption of tooth brushing techniques involving the use of more or less abrasive toothbrushes and pastes.

The use of toothbrushes indeed became widespread after 1885, when mass and industrial production of toothbrushes began following a patent filed by H.N. Wadsworth in 1857 [18].

However, the use of toothbrushes or tools for dental brushing, is documented throughout history in several populations, including Indian, Arab, and Chinese, primarily for oral hygiene as well as ritualistic purposes, as in ancient Rome [19]. Nonetheless, these findings are sporadic, and do not demonstrate a widespread adoption of oral hygiene practices involving the use of tools comparable to toothbrushes.

An interesting phenomenon that has also affected cultural and ethnic groups from the past, is the self-induced sharpening of anterior teeth, both through tools and through thegosis (from the Greek “to go to whet or sharpen,” a term that could be used to describe the sharpening of anterior teeth in specific situations, often guided by social context). This practice is employed both as a ritualistic aspect, and in the reduction of myofascial pain induced by bruxism, to alleviate painful symptoms [20].

The current prevalence of NCCLs in the population varies according to different authors, ranging from 63% [21] to 13% [22], depending on factors such as a diet rich in acids or habits like excessive brushing, with the judgment regarding the role of abfraction still pending.

The consumption of abrasive and hard foods by ancient populations leads, in this way, to the rapid wear of the vertical dimensions of tooth crowns, resulting in a reduction in the flexibility of the teeth in the cervical sites [23].

The purpose of this systematic review was to investigate, whether the scientific literature describes the presence of NCCLs in ancient populations, and to conduct a quantitative analysis with meta-analysis, in order to determine if there is a different or similar prevalence of these lesions before the introduction of toothbrush. This hypothesis, would further support abfraction as the mechanism behind NCCLs onset.

## Materials and methods

### Protocol

The planning of this systematic review and meta-analysis was performed according to the recommendations of the Cochrane Handbook for Systematic Reviews of Interventions [24]. The manuscript was prepared following the indications of the PRISMA (Preferred Reporting Items for Systematic Reviews and Meta-Analysis) [25]. The protocol was registered on the PROSPERO platform (International Prospective Register of Systematic Reviews), with number CRD42023455735, before proceeding with item selection.

### Eligibility criteria

All studies that reported data on the presence of NCCLs on teeth in ancient or prehistoric human skulls, and clearly distinguished between occlusal dental wear, carious lesions, and non-carious lesions, were considered potentially eligible. The PICO question was as follows: what is the proportional number of teeth with NCCLs in ancient or prehistoric skulls compared to modern era skulls? (P) participants (ancient or prehistoric skulls), (I) intervention (presence of NCCLs), (C) control (None), and (O) outcome (proportions of teeth with NCCLs to total teeth in ancient skulls).

The inclusion criteria were as follows: studies that reported data on the presence of carious and non-carious lesions on the teeth cervical margin in ancient and prehistoric skulls, and provided the exact number of teeth affected by the condition compared to the total sample.

The exclusion criteria were: studies that did not report data on NCCLs in ancient skulls, or did not differentiate between occlusal dental wear, carious lesions, and non-carious cervical lesions;

A language filter was applied during the search to exclude reports with abstracts or full texts available in a language other than English.

To minimize publication bias, a detailed search of individual records, as well as references from previous reviews, Grey literature, textbooks, proceedings, doctoral theses, and conference abstracts, was conducted.

The specific search for these sources was initially conducted through search engines such as Google Scholar and ScienceDirect, aiming to identify doctoral theses, textbooks, conference proceedings and preprints, all of which are indexed on these platforms. Subsequently, using keywords, relevant records on the topic of NCCLs and ancient skulls were identified. Doctoral theses or book texts containing potentially suitable data were included; however, if they lacked adequate data but were relevant or offered interesting yet incomplete information, the information source cited in the text was examined through the bibliographic reference, which referred to an article or study that may not have been previously included but provided useful data and information.

This approach aimed to identify the maximum number of reports, with the source of information not being considered as an exclusion criterion.

### Sources of information, Research and Selection

The studies were identified through bibliographic searches of electronic databases by two authors (M.D. and D.S.). Restrictions were applied to the language of publication, and articles not in English were excluded. The bibliographic search was conducted on the databases of PubMed, Scopus, and the Cochrane Library. The last literature search was conducted on August 16, 2023. Additionally, a search of Grey literature was also performed by consulting Google Scholar, Science Direct, and Open Gray. The bibliographic sources related toprevious systematic reviews on the topic were also investigated, and special attention was given in order to identify the highest number of reports on the subject.

The following search terms were used for searching the databases: ancient SKULL AND (dental OR tooth), NCCL, Abfraction.

The following search terms were used on PubMed: Search: Ancient Skull AND (dental OR Tooth OR enamel) Sort by: Most Recent.

(“ancient“[All Fields] OR “anciently“[All Fields] OR “ancients“[All Fields]) AND (“skull“[MeSH Terms] OR “skull“[All Fields] OR “skulls“[All Fields] OR “skull s“[All Fields]) AND (“dental health services“[MeSH Terms] OR (“dental“[All Fields] AND “health“[All Fields] AND “services“[All Fields]) OR “dental health services“[All Fields] OR “dental“[All Fields] OR “dentally“[All Fields] OR “dentals“[All Fields] OR (“teeth s“[All Fields] OR “teeths“[All Fields] OR “tooth“[MeSH Terms] OR “tooth“[All Fields] OR “teeth“[All Fields] OR “tooth s“[All Fields] OR “tooths“[All Fields]) OR (“dental enamel“[MeSH Terms] OR (“dental“[All Fields] AND “enamel“[All Fields]) OR “dental enamel“[All Fields] OR “enamel“[All Fields] OR “enamels“[All Fields] OR “enamel s“[All Fields] OR “enameled“[All Fields] OR “enameling“[All Fields] OR “enamelling“[All Fields]))Translations Ancient: “ancient“[All Fields] OR “anciently“[All Fields] OR “ancients“[All Fields] Skull: “skull“[MeSH Terms] OR “skull“[All Fields] OR “skulls“[All Fields] OR “skull’s“[All Fields] dental: “dental health services“[MeSH Terms] OR (“dental“[All Fields] AND “health“[All Fields] AND “services“[All Fields]) OR “dental health services“[All Fields] OR “dental“[All Fields] OR “dentally“[All Fields] OR “dentals“[All Fields]Tooth: “teeth’s“[All Fields] OR “teeths“[All Fields] OR “tooth“[MeSH Terms] OR “tooth“[All Fields] OR “teeth“[All Fields] OR “tooth’s“[All Fields] OR “tooths“[All Fields] enamel: “dental enamel“[MeSH Terms] OR (“dental“[All Fields] AND “enamel“[All Fields]) OR “dental enamel“[All Fields] OR “enamel“[All Fields] OR “enamels“[All Fields] OR “enamel’s“[All Fields] OR “enameled“[All Fields] OR “enameling“[All Fields] OR “enamelling“[All Fields].

An update of the PubMed keywords was performed on 15 January 2024, with the addition of the following key words:

Search: (“archeological” OR “osteoarcheological” OR “medieval” OR"15th century” OR “14th century”) AND dental: (“archeological“[All Fields] OR “osteoarcheological“[All Fields] OR “medieval“[All Fields] OR “15th century“[All Fields] OR “14th century“[All Fields]) AND (“dental health services“[MeSH Terms] OR (“dental“[All Fields] AND “health“[All Fields] AND “services“[All Fields]) OR “dental health services“[All Fields] OR “dental“[All Fields] OR “dentally“[All Fields] OR “dentals“[All Fields]).

Translations, dental: “dental health services“[MeSH Terms] OR (“dental“[All Fields] AND “health“[All Fields] AND “services“[All Fields]) OR “dental health services“[All Fields] OR “dental“[All Fields] OR “dentally“[All Fields] OR “dentals“[All Fields].

However, for the Scopus platform, the following search terms and criteria were used:

TITLE-ABS-KEY (ancient AND skull AND (dental OR tooth OR enamel)).

Duplicates were deleted using EndNote and manually. The identified reports were subject to an independent evaluation and review, by two separate investigators (M.D. and D.S.). Evaluation of potentially eligible articles was based on evaluation of titles and abstracts, while full content was reviewed for the inclusion in the systematic review. In addition, any discrepancies were resolved by a third reviewer (A.B.). At the end of the articles selection, a final search was conducted to identify further studies among the references of the included studies.

### Data Collection process and data characteristics

The type of data and information to be extracted were previously determined by the two authors responsible for screening the articles. Once the categories of interest had been delineated, the two authors independently conducted data extraction from each selected article. This approach was adopted in order to reduce the risk of mutual influences and ensure greater objectivity in data extraction. The results of this phase were subsequently recorded into separate tables, structured in a coherent way, to facilitate comparison and subsequent analysis.

The comparison between the two author’s tables was carried out accurately, in order to identify any discrepancies lead to a shared view of the data. This methodology has proven to be effective in reducing the risk of bias in reporting, ensuring a more objective interpretation of the overall results.

The data that were extracted from the articles include the first author, the year of publication, the country that conducted the study, the number of skulls, the number of teeth, the presumed historical period, the position of the site of the discovery of the findings, the presumed mean age, the presumed sex, the presence of NCCLs, the lesion type or form.

### Risk of bias

Tools such as STROBE (STrengthening the Reporting of OBservational studies in Epidemiology), are available to standardize the reporting of observational studies, but they prove inefficient for assessing the risk of bias in the archaeological and anthropological context. No validated assessment tool, is currently available in the literature to address the methodological quality of studies reporting archaeologically skulls and, consequently, the risk of bias associated with them. Existing tools are designed for evaluating observational or intervention studies in the medical scientific literature, and are not applicable to this specific type of study, due to their potential unique sources of bias related to the nature of the artifact (skeleton/skulls), such as representativeness, preservation, aging, skeleton sex determination, and anthropological origin. Moreover, specific guidelines in the fields of archaeology and anthropology, should be regarded as appropriate methodologies to adopt during skeletal excavation procedures [26, 27] .

The sole tool for evaluating studies dealing with excavated human skeletons was the study conducted by Rajbhoj et al. [28], developed using the Delphi technique through questionnaires among experts in the field, including anthropologists, archaeologists, medical professionals, epidemiologists, dentists, and forensic experts.

Thus, our assessment of the risk of bias was carried out using a scale based on the “Assessment of methodological quality of articles with excavated human skeletons” [28], which is established in 17 items.

### Summary measures, summary of results

Individual study results were extracted and documented in tables, while aggregated data were incorporated and visually represented through figures such as the forest plot, accompanied by corresponding numerical values depicting a ratio: Events (NCCLs) / Total teeth for each individual study. Additionally, the final aggregated value will be reported, accompanied by confidence intervals and heterogeneity indices like the Higgins index (I_2_).

For the meta-analysis, specifically for calculating pooled ratios, the Open Meta-Analyst software version 10 was employed.

## Results

### Selection of studies

The research question that guided the study selection was as follows: “what was the presence of non-carious cervical lesions (NCCLs) in ancient skulls?”. Therefore, the studies to be included were those reports that provided information on the number of teeth with NCCLs compared to the total number of teeth and the number of individuals/skulls with at least one NCCLs compared to the total number of individuals/skulls.

The research phase was conducted by consulting and extracting the bibliographic references on two databases, SCOPUS (325 records) and PubMed (420 records) and on a Cochrane library (387 trials), to which was added the Gray literature analysis,through Science direct (*n* = 12,361), Google scholar (*n* = 8,110) and OpenGrey (http://www.opengrey.eu, DANS EASY Archive) (*n* = 4) providing a number of 21,607 records. Scopus and PubMed references were uploaded to the EndNote X8 and duplicates removed using software, while duplicates not identified by EndNote were manually removed.

**Fig. 1:**
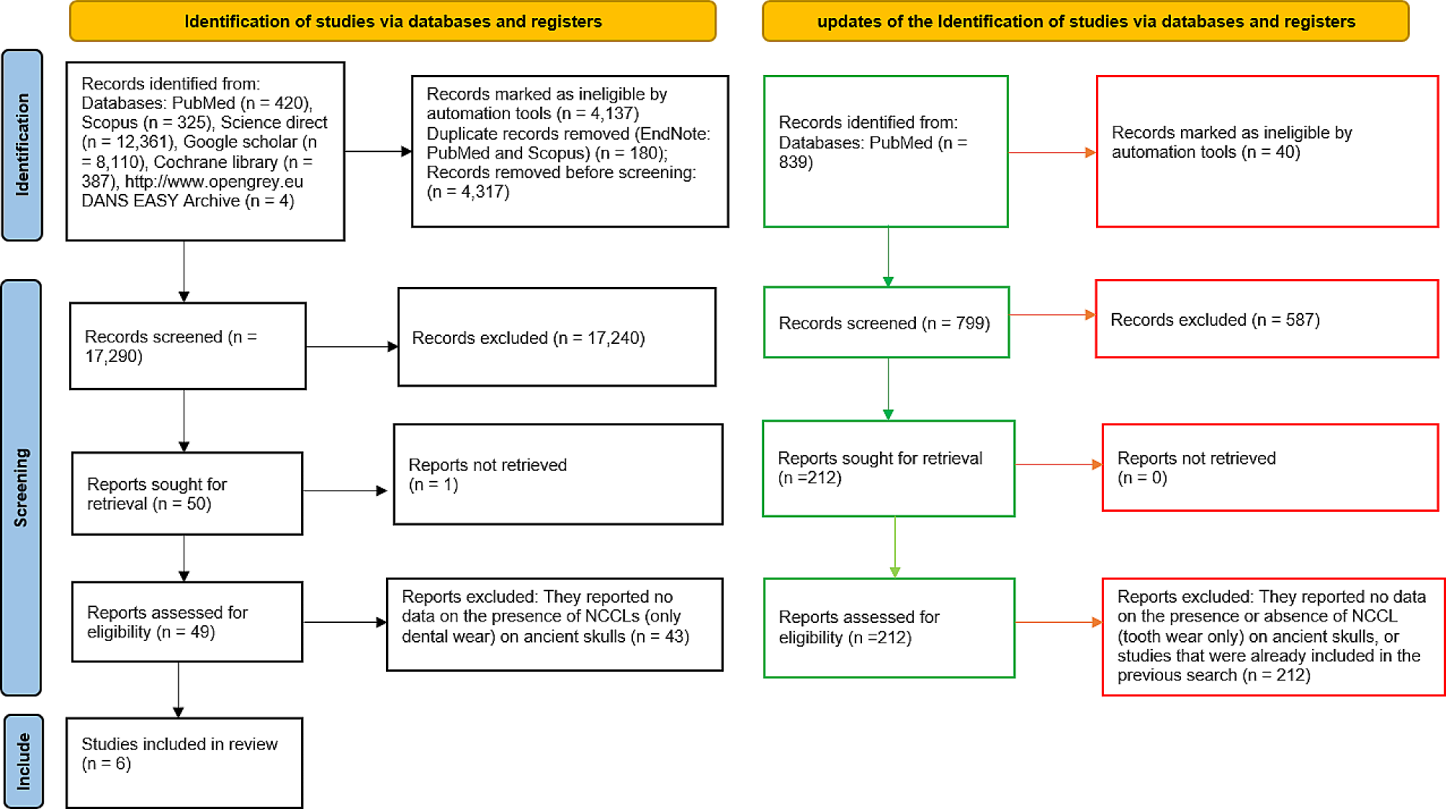
Entire selection and screening procedures are described in the PRISMA flowchart. The red and green boxes show the results of the research conducted on 15 January 2024, using the keyword : (“archeological” OR” osteoarcheological” OR “medieval” OR “15th century” OR “14th century”) AND dental

After reviewing the title and abstract of each record, a total of 50 potentially eligible articles were identified. Following the selection process, 6 articles were included for quantitative evaluation. Additional searches were conducted within previous systematic reviews, but no further studies were identified for inclusion in the review (Fig. 1).

Was obtained a number of records equal to 839, which were screened by the two authors in search of any clinical studies to be included, and the results of this selection were highlighted in Fig. 1. Screening of these additional records, did not result in the inclusion of further studies that had already been considered.

### Data characteristics

The articles included in the review were: Aubry et al. 2003 [29], Aaron, 2004 [30], Urzúa et al. 2015 [31], Bondioli et al. 2012 [32], Kieser et al., 2001 [33] and Ritter et al., 2009 [34].

The extracted data are presented in Table 1, encompassing information concerning the primary author, publication year, study’s country of origin, count of skulls, number of teeth, presumed historical period, location of the discovery site, estimated average age, inferred sex, presence of NCCLs, and lesion type or form.

**Table 1:** Characteristics and data of the studies included in the systematic review; N (number), m (male), f (female), U (Unknown), AD (Anno Domini), BC (Before Christ), \ (No data, data not reported)

First autor, data	Country	Site and period of the finds	Sex	N ancient skulls	N of teeth examined	N of NCCL	N Ancient skulls with NCCL	N of modern skulls	N of teeth examined, NCCL	N of modern skulls with NCCL’	Professional or academic training of observers (diagnostic skills)
Kieser et al., 2001 [33]	New Zealand	pre-contact New Zealand Maoris ^d^	50 (27 m, 23 f)	50	\	\	0	\	\	\	Dentist, Orthodontics
Aubry et al. 2003 [29]	France	Roaix, (2150–2090 BC) Saint-Pierre de l’Almanarre, (1100–1300 AD), Notre-Dame du Bourg (1300–1400 AD)	Roaix 76 (14 m, 12 w, 50 U), Saint-Pierre de l’Almanarre 100 (60 m, 40 f), Notre-Dame du Bourg 56 (19 m, 9 f, 28 U)	259	3927	0	0	238	6,1451\277 NCCLs	62	Anthropologist, Dentist
Aaron, 2004 [30]	USA	Northern Illinois (11th century), South Dakota (17th century)	Northern Illinois 50 (26 m, 24 f), South Dakota 50 (25 m, 25 f)	100	2553	0	0	198	3,524 teeth in the modern skulls, 57 NCCLs	19	Dentist, Periodontology
Ritter et al., 2009 [34]	USA	AD 650 to the late 19th century: 23 Labrador, Canada ( native americans 500 years old); 23 Merida, Mexico (late 19th century); 13 from the Mimbres Valley sites in New Mexico (AD 650–950); 23 Madisonville, Ohio, USA (AD 1275–1640). 22 from Mistihalj, Montenegro (15th and 16th centuries)	labrador 23 (7 m, 16 f), Merida 23 (14 m, 9 f), New Mexico 13( 10 m, 3 f), Ohio 23 (12 m, 11 f), Montenegro 22 (15 m, 7 f)	104	\	\	7	\	\	\	Dentist, Archaeologist
Bondioli et al. 2012 [32]	Italy	Pakistani Neolithic site of Mehrgarh	\^c^	225	3577^b^	123	10	\	\	\	Anthropologist, Archaeologist, Biologist
Urzúa et al. 2015 [31]	Chile	San Pedro de Atacama oases are located in the northeast of Chilete de Chile, 400–1300 AD	\	67	1521^a^	0	0	\	\	\	Dentist

^a^The number of teeth was extrapolated from the average number of teeth per individual multiplied by the number of individuals

^b^The number concerns only the permanent teeth with the deciduous ones would be 3880

^c^The Skulls with NCCL are male (10 in total)

^d^The place where the remains (Skulls) were found is not specified in the manuscript (New Zealand?)

To provide further clarification of the six included studies, four studies, despite explicitly examining the presence of NCCLs as outlined in the materials and methods sections and reported in the results, did not identify the existence of NCCLs in ancient skulls

### Risk of Bias

The evaluation of the risk of bias was carried out using a scale based on the “Assessment of the methodological quality of articles with excavated human skeletons”, which is based on 17 items. (1, clearly stated aim; 2, primary outcome; 3, racial/ethnic distribution; 4, condition of the sample; 5, sample preservation; 6: age-at-death; 7, method for age-at-death estimation; 8, sex distribution; 9: method for sex determination;10, time period of the sample; 11, dating method; 12: method(s) of measurement; 13, statistical tests; 14, outcome reporting; 15, blind testing; 16, baseline equivalence of groups; 17: presence of other bias;) For the evaluation of the risk of bias some items were combined for practical reasons and similarity of the risk of bias in a single evaluation: Items 6 (age-at-death) and 7, (method for age -at-death estimation); Items 8 (sex distribution) and 9 (method for sex determination); Items 10 (time period of the sample) and 11 (dating method); while items 17 were considered to be evaluated separately by including any problems in the limits section of the review and not implemented in Table 2. Therefore the evaluation was made on a total of 13 items.

Baseline equivalences were extrapolated, and a value was assigned for each manuscript, from 0 to 2 (Table 2).

The risk of bias was assessed as acceptable for all studies included in the meta-analysis, and the main critical issues for each manuscript are reported below.

**Table 2:** Risk of bias, Score interpretation: Zero (0) if the variable is not reported in the article, one (1) if the variable is reported but inadequate, Two (2) if the variable is reported and adequate (\) not applicable

	Items	Aaron, 2004 [30]	Aubry et al. 2003 [29]	Urzúa et al. 2015 [31]	Bondioli et al. 2012 [32]	Kieser et al., 2001 [33]	Ritter et al., 2009 [34]
Clearly stated aim	The aim of the study is essential to make an unbiased assessment of the findings of studies with excavated human skeletons.	2	2	2	2	2	2
Primary outcome	The primary outcome is essential to make an unbiased assessment of the findings of studies with excavated human skeletons.	2	2	2	2	2	2
Ancestry/ethnicity distribution	Reporting the ancestry/ethnicity of the sample is essential to determine possible outcome bias in studies with excavated human skeletons.	2	2	2	2	2	2
Condition of the sample	Reporting the condition of the sample is essential to make an unbiased assessment of the findings in studies with excavated human skeletons.	1	2	2	2	1	2
Sample preservation	Reporting the preservation methods used on the sample is essential to make an unbiased assessment of the findings in studies with excavated human skeletons.	1	2	2	2	1	2
Age-at-death distribution	Reporting of the age-at-death distribution of the sample and the method for its estimation is important to make an unbiased estimation of the outcome of studies with excavated human skeletons.	2	2	2	2	0	2
Sex distribution	Reporting of the sex distribution of the sample and the method for its estimation is important to make an unbiased estimation of the outcome of studies with excavated human skeletons.	2	2	0	1	2	2
Time period	Reporting of the time period of the sample and the dating method used is important to make an unbiased estimation of the outcome of studies with excavated human skeletons.	2	2	2	1	1	2
Method of measurement	Reporting the method(s) of measurement is important to make an unbiased estimation of the outcome of studies with excavated human skeletons.	2	2	2	2	2	2
Statistical test	Reporting appropriate statistical tests in accordance with the study type and primary outcome of the study is essential to make an unbiased estimation of outcome of studies with excavated human skeletons.	2	2	2	1	2	2
Selective or incomplete reporting	Selective or incomplete outcome reporting may bias the findings of studies with excavated human skeletons.	2	2	2	2	2	2
Blind Testing	Blind Testing of research staff may reduce bias in certain settings of studies with excavated human skeletons.	2	2	\	\	\	\
Baseline equivalence	In comparative studies with excavated human skeletons, reporting the baseline equivalence of the groups to be compared is essential to make an unbiased estimation of the outcome.	2	2	\	\	\	\

- Aaron, 2004 [30]. Condition of the sample: (1) there is no adequate description of the samples taken into consideration, and in fact only a generic qualitative and non-quantified description of the state of the skulls is present in the discussion and not in the materials and methods or in the results. Sample preservation: (1) Reports that the samples are preserved at the Museum of Natural History in Washington.
- Kieser et al., 2001 [33]. Condition of the sample: (1) does not provide data on the state of conservation of the skulls but nevertheless reports fundamental data strictly related to dental wear, Age-at-death distribution: (0) are not reported information relating to the age of the skulls or to the evaluation method used to determine it; Sample preservation: (1) ) Reports that the samples that are part of the collection of the Department of Anatomy and Structural Biology at the University of Otago, Time period: (1) the estimated dating of the skulls is not explicitly reported in the manuscript but reference is made only to a period prior to contact with Europeans.
- Urzúa et al. 2015 [31]. Sex distribution: (0) there are no data relating to sex and the estimation method.
- Bondioli et al. 2012 [32]. Sex distribution: the evaluation and methodology to determine sex was performed and described but only the sex data of the subjects who presented NCCL (Men only), are reported in the manuscript. Statistical test (1): a real statistical analysis has not been performed in fact there are only data expressed in percentages.

### Meta-analysis

The meta-analysis of the data was conducted using version 10 of Open Meta-Analyst,and the data represented through forest plots.

**Fig. 2:**
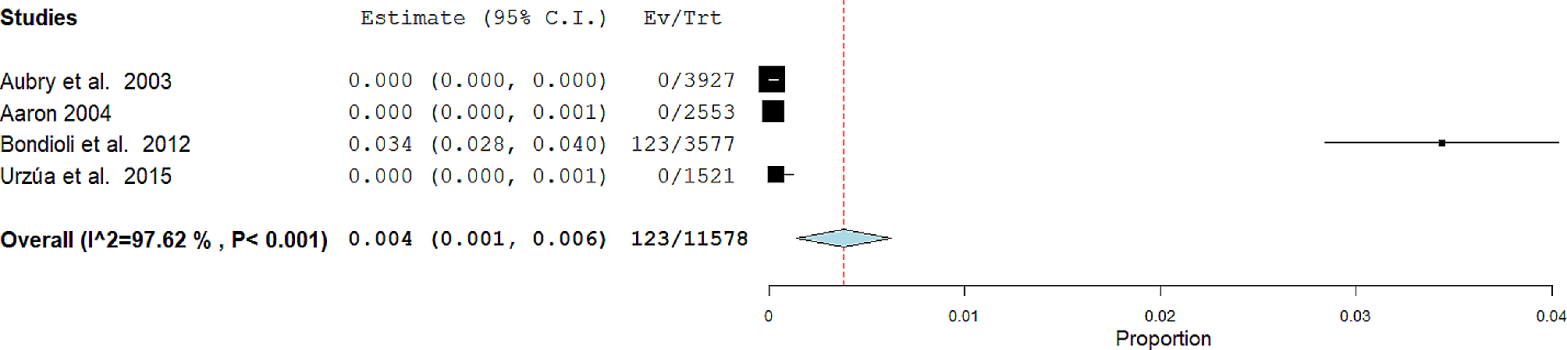
Binary random effects model metric; proportion: 0.004; C.I. (Confidence Interval): (lower bound) 0.001(upper bound) 0.006; *p*-value 0.002; Standard error (SE): 0.001; Q = Q statistic; df = degrees of freedom; I2 (I^2) = Higgins heterogeneity index, I2 < 50%, heterogeneity low; P = *p* value; heterogeneity (Het.): tau^2: 0.000 Q (df = 3) 126.062, Het. *p*-value: < 0.001, I^2: 97.62; The graph of each study shows the first author and the date of publication as well as the measurement of the number of NCCL on the total teeth and the relative proportion: with the confidence intervals reported. The final value with the relative confidence intervals is expressed in bold. The dashed red line shows the position of the average value, and the light blue diamond shows the measure of the average effect

The first meta-analysis was conducted on the ancient skulls NCCLs presence in teeth. There were 4 studies included in this meta-analysis (Aaron, 2004 [30], Aubry et al. 2003 [29], Urzúa et al. 2015, Bondioli et al. 2012 [32]), random effects were applied according to DerSimonian and Laird, by calculating the ratio between teeth with NCCLs on the total number of teeth examined. The final ratio was 123\11,578 (Fig. 2).

The second meta-analysis, calculated the ratio of skulls that presented NCCLs compared to the total number of skulls examined. The studies included in this meta-analysis were 6 (Aubry et al. 2003 [29], Aaron, 2004 [30], Urzúa et al. 2015 [31], Bondioli et al. 2012 [32], Kieser et al. 2001 [33], and Ritter et al. 2009 [34],).

**Fig. 3:**
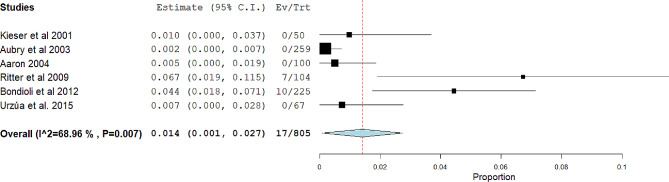
Binary random effects model metric; proportion: 0.014; C.I. (Confidence Interval): (lower bound) 0.001(upper bound) 0.027; *p*-value 0.036; Standard error (SE): 0.007; Q = Q statistic; df = degrees of freedom; I2 (I^2) = Higgins heterogeneity index, I2 < 50%, heterogeneity low; *P* = *p* value; heterogeneity (Het.): tau^2: 0.000 Q (df = 5) 16.111, Het. *p*-value: 0.007, I^2: 68.964; The graph of each study shows the first author and the date of publication as well as the measurement of the number of skulls with NCCLs on the total skulls, and the relative proportion: with the confidence intervals reported. The final value with the relative confidence intervals is expressed in bold. The dashed red line shows the position of the average value, and the light blue diamond shows the measure of the average effect

Also in this case, random effects were applied according to DerSimonian and Laird, and the final ratio was 17 skulls on a total of 805 (Fig. 3).

## Discussion

### Summary evidence

The authors conducted a systematic literature review with a meta-analysis to identify the prevalence of in the ancient population through the analysis of skulls. At the end of the systematic review selection phase, 6 studies that had investigated the presence of NCCLs, were identified. Among these, only 2 studies had identified these peculiar lesions in ancient skulls [29, 30]. In particular, the total number of teeth examined was 11,578, and the number of skulls examined was 805; NCCLs, were found in a total of 17 subjects.

The purely quantitative analysis of these data appears to indicate the presence of NCCLs even in periods when there was no industrial distribution of toothbrushes. This suggests an etiopathogenetic mechanism determined by abfraction, in which components constituted by non-axial occlusal forces alone, can lead to the onset of lesions. However, this assessment of the results does not take into account that, even in pre-industrial times, there were tools capable of causing abrasions, in the cervical areas of the teeth, both for oral hygiene, ornamental, or ritual purposes, [35].

In particular, by analyzing the manuscripts, were identified only 2 studies that report the presence of NCCLs: Bondioli et al. [32] and Ritter et al. [34]. The presence of NCCLs in ancient skulls does not clarify the etiology of the lesions, due to the difficulty in determining their cause. It is not possible to unequivocally attribute them to abfraction phenomena, as abrasive phenomena cannot be ruled out. In fact, Bondioli et al. [32], report that the responsible cause of the NCCLs discovered at the Neolithic site of Mehrgarh is challenging to identify clearly.many NCCLs described in this study, were not limited to the cervical area of the tooth, but more extended, involving a significant portion of the root and crown [32]. Furthermore, it is important to note that in conjunction with these lesions, there was an absence of high levels of periodontal disease (this is partly in agreement with Grippo’s observations on the pathological mechanism of abfraction) [14]. The lesions had a “C” shape and primarily involved the buccal surface of the tooth (114 cases) and in some instances, to the lingual surface as well (9 in total). Additionally, a distinctive feature of these lesions, was the presence of thin horizontal striations across the entire surface of the tooth and the affected root.

The cervical lesions described exhibited differences from modern ones, as reported by Aubry [32]; they present a “saucer-shaped” appearance rather than a “wedge-shaped” one, appearing to be more widely distributed around the dental arch and affecting individuals, presumably males, above the age of 40. Occlusal wear levels were high, and the presence of carious tooth lesions was extremely rare [29].

In the study conducted by Bondioli et al. [32], corrosion and abfraction were not considered as etiopathological mechanisms of NCCLs. Instead, these lesions were attributed to manipulative abrasive phenomena [32].They also suggested, as a possible cause, the use of labrets, as indicated by Torre [32], in a finding from the pre-Columbian Chilean period (400–900). In this case, the shape of the lesions was very similar to those described by Bondioli et al. [32], still “saucer-shaped,” for the use of quartz labrets [36]. However, the lesions are generally reported in a similar manner in other historical findings [37–39], as previously mentioned [32].

On the other hand, Ritter et al. [34], reports the presence of NCCLs in 7 skulls, indicating that these lesions were not prevalent among similar populations and were found predominantly in Mexicans (26%). The type of lesions described by Ritter’s group is consistent with abfraction, as also evident from the images published in the study, because the lesions exhibited a wedge-shaped form, typical of non-carious lesions. As for the origin of these lesions, Ritter provides slightly different reasons compared to Bondioli et al. [32].

In particular, his group reports the presence of NCCLs in 6 skulls without specifying the exact number of lesions [32]. These skulls belonged to a late 19th-century population in the Yucatan region of Mexico, which had at least partially inherited the Mayan culture [34].

Higher levels of occlusal wear in New Mexico, coupled with the loss of occlusal anatomy and a significantly reduced crown-to-root ratio, lead to a decrease in non-axial occlusal forces in their horizontal component, consequently reducing stress in the cervical area and becoming a limiting factor in the development of NCCLs [34].

Higher levels of occlusal wear, lower than in other groups (Labrador, New Mexico, Ohio, and Montenegro), and the consumption of peculiar foods, such as the use of tomatoes and fermented grains, could theoretically have contributed to the formation of NCCLs in the Mexican population [34].

In prehistoric populations, excessive occlusal wear was a common cause of tooth loss [40], especially before the widespread occurrence of dental caries with the introduction of industrial sugar production [29].

Urzúa et al. [31], report data on dental occlusal wear in 66 individuals and totally zero NCCLs in a population from northeastern Chile between the age from 400 to 1400, confirming with this study, a pattern of dental wear determined by a non-acidic diet [31].; the abrasive nature of food is indicated as the main cause of this wear [33].

Dietary factors appear to be the key to understanding the presence of NCCLs in the modern era, with a reduced incidence of this specific lesions in ancient times [41].

The type of diet followed by populations in a given location and time period, could therefore influence the incidence of NCCLs, either by discouraging their onset through occlusal tooth surface wear, or by favoring it due to a potential acidic component that causes erosion in the cervical areas of the teeth [42].

Another factor that may have influenced the incidence of NCCLs in ancient skulls is the abrasive component, not necessarily caused by toothbrush use, but by the use of labrets [36] or other objects derived from intentional manipulations or ritual purposes. Abrasion in the cervical area of the tooth caused by the rubbing of objects or artifacts already had the capacity to generate NCCLs, and when combined with an acidic diet and soft foods, the component represented by non-axial occlusal forces becomes crucial in explaining the explosion of NCCLs cases in the 20th century. The data is clear, as taking into consideration both modern and ancient skulls, Aroon’s data report cases of NCCLs only for modern skulls [30], in agreement with Aubry et al.‘s findings [29].

From the data available in the literature, it emerges that the presence of NCCLs has also been identified in 17 ancient skulls from a total of 805, but with a lower prevalence compared to the modern population: from the data of 2 included studies, compared the lesions also to the modern population, there are 81 skulls with NCCLs (436 in total).

The analysis of documents included in the review suggests as the main hypothesis that the reduced presence of these lesions in ancient populations is due to extensive and widespread wear of the occlusal surface, caused by abrasive phenomena associated with the consumption of extremely abrasive foods, such as hard foods or those containing lithic components. Furthermore, ancient populations followed a diet poor in terms of acid substances, which may have contributed to reducing the erosive nature in the formation of non-carious cervical lesions. Indeed, in populations presumed to have an acid component in their diet (such as tomatoes and fermented wheat), and utilizing a different cereal grinding technique, the presence of NCCLs has been documented. However, these lesions exhibit typical wedge-shaped forms (Mexico), indicative of abfraction rather than acid erosion. While acidity may have contributed to the formation of these lesions, their characteristic shape suggests the presence of additional concurrent etiological factors [34].

On the other hand, in the post-industrial era, the presence of softer and more acid foods, along with the introduction of toothbrushes, has contributed to the increase of these lesions, especially concerning the genesis of NCCLs determined by abfraction [43]. Therefore, in the modern era, three main factors contribute to the formation of these lesions, as indicated by the analysis of ancient skulls and dietary habits:

- 1-) The introduction and extensive use of artifacts, such as toothbrushes, for example, whose improper use causes abrasion in the cervical area of the teeth (it has also been documented that the use of labrets has resulted in cervical wear due to abrasion in historical artifacts) [44];
- 2-) A softer and non-abrasive diet for the occlusal surface, with cusps preserved and a greater potential for non-axial occlusal forces to act on the cervical enamel zones of the tooth [43];
- 3-) A higher presence of an acidic or acidophilic diet, which favors the occurrence of erosive phenomena [45].

### Meta-analysis

The meta-analysis of data depicted in the two forest plots (Figs. 2 and 3) first highlights that the majority of studies report zero NCCLs in ancient skulls. Specifically, only 2 out of 6 studies provide data, albeit in a relatively limited number compared to the modern population (Table 2, columns 10 and 11). The second aspect that emerges is the high heterogeneity determined by the inconsistency index exceeding 50% in both meta-analyses.

In the first meta-analysis, despite an overlap of confidence intervals from 3 out of 4 studies, the Bondioli et al. [32] study likely contributes to increased heterogeneity (I2: 97.62). Subgroup and sensitivity analyses to identify sources of heterogeneity prove impractical due to the low number of included studies. In the second meta-analysis, we observe slightly lower heterogeneity than the first (I2: 68.964), with data from Bondioli et al. [32] and Ritter et al. [34], overlapping in confidence intervals, and the remaining 4 studies partially overlapping in sample sizes and NCCLs presence (0).

The final data, as extensively reported in the results section, indicate 17 skulls with NCCLs out of a total of 805, with a percentage of 2.11%. Compared to the data from modern populations included in the study, which stands at 18.58% (Table 2), these figures are notably lower. Regarding teeth affected by NCCLs, the percentages are 1.06%, with 23 of 11,578 total teeth in ancient skulls.

### Limitations of the review

The main limitation of this systematic review with meta-analysis is the low number of included reports. Specifically, only six studies explicitly addressed Non-Carious Cervical Lesions (NCCLs), as indicated in the materials and methods section or mentioned in the results. The inclusion of studies that did not make reference to NCCLs, lesions related to abrasion, or erosive phenomena affecting the cervical surface of teeth could have introduced bias into the data. On the other hand, efforts were made to mitigate publication bias by searching for reports in the grey literature that might be challenging to retrieve from major databases.

Furthermore, certain studies included in the analysis failed to provide the count of skulls with Non-Carious Cervical Lesions (NCCLs), instead only presenting the total number of teeth affected by the lesions.

However, the limitations of this systematic review include difficulties in clarifying the etiological origin of the lesions, as it is not possible to exclude an abrasive or erosive cause simply related to excessive occlusal loads.

A further limitation of the review is that the nature of these lesions is challenging to diagnose using the available evidence. In fact, some historical and prehistoric communities utilized various devices for oral hygiene, including roots or even tree branches [46]. These instruments could lead to traumatic injuries in the gingiva-periodontal area, resembling wedge-shaped abrasion lesions caused by brushing trauma.

One aspect that should be considered, potentially posing a limitation, is the ability of examiners in individual studies to distinctly identify the presence of non-carious cervical lesions (NCCLs) in ancient skulls and make accurate diagnoses. This issue appears to be clearly addressed by academic dentists in four studies. However, in two studies, it is not apparent among the authors or in the materials and methods section that dentists conducted the examination Bondioli et al. 2012 [32], Aubry et al. 2003 [29]: only modern subject series patients from in three dental practices, instead, it was carried out by anthropologists or archaeologists. Nevertheless, in our view, this does not necessarily indicate a diminished capacity to identify dental lesions. Anthropologists and archaeologists are adept at interpreting signs, such as tooth wear, and more broadly, pathological conditions, enabling them to reconstruct the subject’s past and habits. In contrast, less experienced dentists examining ancient skulls might mistakenly identify lesions to hard tissues that occurred after burial or resulted from sample deterioration.

Moreover, the limited number of included studies reduces the possibility of conducting subgroup and sensitivity analyses based on populations or periods of interest, thus maintaining a high degree of data heterogeneity (I2 68%).

## Conclusions

In conclusion,is possible to speculate that NCCLs were present in some populations with prevalences certainly much lower than those observed today, but the etiological causes for the scarcity of evidence cannot necessarily be attributed to Abfraction mechanisms. Furthermore, although there are reports of NCCLs occurring before toothbrushes discovery, this does not necessarily imply that abrasion as etiologic factor. Currently, there is insufficient data to support such a claim. Taking into account the limitations and the multitude of variables, impossible to be determined in ancient skulls, connecting NCCLs to an occlusal etiology is challenging to demonstrate if it truly exists and is inherently speculative.

Instead, hypothetical determinants appear to be related to abrasive aspects associated with diet, influencing occlusal wear and reducing non-axial occlusal forces that would lead to the presence of NCCLs.

The lack of clarity regarding the prevalence and etiological causes of NCCLs in ancient populations is attributed to the limited availability of studies, reporting or finding the presence of such lesions. Additionally, the absence of data on the etiology of NCCLs found in ancient skulls contributes to the difficulty in drawing definitive and clear conclusions about the causes of these lesions when they are identified. Given this limited evidence, further research are needed to deepen the understanding of the causes of NCCLs in ancient skulls.

## Data Availability

All data generated or analysed during this study are included in this published article.
